# Diabetic Autonomic Neuropathy Among Patients With Diabetes in Rural Warangal in India: A Cross-Sectional Study

**DOI:** 10.7759/cureus.115194

**Published:** 2026-08-25

**Authors:** Sneha Simon, Balabaskaran S, Shravani M, Nirmala Devi B, Punam Kumari Jha

**Affiliations:** 1 Community Medicine, Sri Venkateshwaraa Medical College Hospital and Research Centre, Puducherry, IND; 2 Community Medicine, Government Medical College, Vikarabad, IND; 3 Community Medicine, Osmania Medical College, Hyderabad, IND; 4 Community Medicine, Government Medical College, Narasempet, IND

**Keywords:** compass 31 questionnaire, diabetic autonomic neuropathy, prevalence, rural-based population, type 2 diabetes mellitus

## Abstract

Background

Diabetic autonomic neuropathy (DAN) is a common but frequently underdiagnosed complication of diabetes mellitus that affects multiple organ systems and contributes to increased morbidity and mortality. Early identification using simple screening tools is essential, particularly in resource-limited primary care settings. However, community-based data on DAN from rural India remain limited.

Objectives

The objective of this study is to estimate the proportion of screen-positive DAN and identify factors associated with its occurrence among individuals with type 2 diabetes mellitus in rural Warangal, Telangana.

Methods

A community-based cross-sectional study was conducted among 400 adults aged ≥30 years with previously diagnosed type 2 diabetes mellitus residing in the rural field practice area of Kakatiya Medical College, Warangal, during 2020-2021. Participants were selected through simple random sampling from the Non-Communicable Disease (NCD) register. Data were collected using a pre-tested semi-structured questionnaire. DAN was screened using the validated Composite Autonomic Symptom Score (COMPASS-31), with a score ≥17 indicating DAN. Descriptive statistics, binary logistic regression, and multivariable logistic regression analyses were performed to identify independent predictors. A p-value <0.05 was considered statistically significant.

Results

The prevalence of diabetic autonomic neuropathy was 21.0% (84/400). On multivariable logistic regression analysis, younger age (≤44 years) (adjusted odds ratio (AOR): 3.10), female sex (AOR: 1.72), illiteracy (AOR: 1.85), unemployment (AOR: 1.67), smoking (AOR: 7.95), alcohol consumption (AOR: 5.21), inadequate daily vegetable intake (AOR: 1.64), physical inactivity (AOR: 1.72), and poor glycaemic control (random blood glucose ≥200 mg/dL; AOR: 6.03) were identified as significant independent factors associated with DAN (p<0.05).

Conclusion

Approximately one in five individuals with type 2 diabetes in this rural population had DAN. Lifestyle-related factors, poor glycaemic control, and adverse socioeconomic characteristics were independently associated with DAN. Community-based screening using validated symptom-based tools such as COMPASS-31, along with interventions promoting glycaemic control and healthy lifestyle practices, may facilitate early detection and reduce the burden of diabetic complications in rural settings.

## Introduction

Diabetes mellitus (DM) [1] refers to a group of metabolic conditions characterized by persistently elevated blood glucose levels, resulting from abnormalities in insulin secretion, impaired insulin action, or a combination of both. Prediabetes [2] is considered a transitional state of dysglycaemia in which blood glucose values are higher than normal but have not yet reached the threshold for a diagnosis of diabetes.

In recent decades, diabetes has become a major global public health concern [3] due to its rapidly increasing prevalence and the significant burden of associated complications. It affects individuals across all age groups and socioeconomic strata and is now recognized as a priority non-communicable disease worldwide. Estimates from the International Diabetes Federation [4] indicate that approximately 463 million adults were living with diabetes in 2019, and this number is expected to rise substantially to 578 million by 2030 and nearly 700 million by 2045. A large proportion of this burden is concentrated in low- and middle-income countries. Nearly four-fifths of adults living with diabetes reside in low- and middle-income countries. India contributes a substantial share to the global burden, with an estimated prevalence of 8.9% (approximately 77 million individuals) in 2019 [5], making it one of the countries with the highest number of people living with diabetes.

India accounts for a considerable share of the global diabetes burden, with tens of millions of individuals affected. The rising prevalence in the country [6] has been attributed to factors such as rapid urbanization, changes in lifestyle, reduced physical activity, and dietary transitions. At the regional level, surveys such as the National Family Health Survey-5 [7] have reported a notable prevalence of diabetes among adults in Telangana, with urban populations generally showing higher rates compared to rural populations. However, increasing trends are also being observed in semi-urban and rural areas, indicating a widespread transition.

Individuals of Indian origin are particularly susceptible to diabetes [8] due to a higher tendency for insulin resistance, increased abdominal fat deposition even at lower body mass index, and a greater prevalence of impaired glucose tolerance. These factors contribute to an earlier onset and higher risk of complications.

Diabetes is associated with a range of long-term complications that significantly impact morbidity and quality of life. These complications are broadly categorized into microvascular complications, including neuropathy, nephropathy, and retinopathy, and macrovascular complications such as cardiovascular and cerebrovascular diseases. Among these, diabetic neuropathy [9] is one of the most frequently encountered complications and may affect both peripheral and autonomic components of the nervous system.

Diabetic autonomic neuropathy (DAN) [10] is a serious yet often under-recognized complication resulting from damage to the autonomic nervous system in individuals with diabetes. It can involve multiple organ systems and present with a variety of clinical features, including abnormalities in heart rate control, blood pressure regulation, gastrointestinal function, bladder function, and sexual function. Cardiac autonomic neuropathy [11], a particularly severe form, is associated with an increased risk of cardiovascular morbidity and mortality.

Beyond its clinical implications, diabetes and its complications impose a substantial economic burden [12] on both individuals and healthcare systems, especially in settings where out-of-pocket expenditure constitutes a major component of healthcare financing. The American Diabetes Association [13] recommends early recognition and management of DAN in order to prevent progression and reduce associated complications. Early identification of complications plays a crucial role in reducing disease progression and improving patient outcomes. Although specialized diagnostic procedures such as autonomic function testing and nerve conduction studies are considered standard, their availability is often limited in primary healthcare settings. In such situations, validated symptom-based tools like the Composite Autonomic Symptom Score (COMPASS-31) [14] offer a practical and feasible approach for screening.

Despite the growing burden of diabetes in India, there is a relative lack of data on DAN, particularly in rural populations using standardized screening methods. Early detection through appropriate screening strategies may help in timely intervention and prevention of further complications.

Therefore, the present study was undertaken to screen for DAN among individuals with diabetes in rural Warangal in India. The specific objectives were to estimate the proportion of screen-positive diabetic autonomic neuropathy and to identify the factors associated with its occurrence in this population.

## Materials and methods

Study design

A community-based cross-sectional study was conducted to screen for DAN among patients with diabetes residing in the rural field practice area of Kakatiya Medical College, Warangal, Telangana.

Study setting

The study was carried out in Wardhannapet, the rural field practice area attached to Kakatiya Medical College, Warangal, located in Telangana state, India. Wardhannapet is a tehsil in Warangal district situated approximately 30 km from Warangal city. According to Census 2011, Wardhannapet village had a population of 13,715 with around 3,050 households and covers a geographical area of about 4,144 hectares. The rural field practice area comprises three villages, namely, Wardhannapet, Bollikunta, and Panthini, which are served by primary healthcare facilities and the non-communicable disease (NCD) clinic. These villages were included in the study.

Study population

The study population consisted of known cases of type 2 diabetes mellitus residing in the selected villages of Wardhannapet taluk in Warangal district.

Inclusion criteria

Men and women aged 30 years and above who were previously diagnosed with type 2 diabetes mellitus and residing in the study area were included in the study. Individuals who provided written informed consent to participate in the study were also included.

Exclusion criteria

Individuals who were not permanent residents of the study area were excluded from the study. Patients who were bedridden, severely ill, or those with dementia or cognitive impairment that prevented effective communication were also excluded.

Study duration

The study was conducted over a period of one year during 2020-2021.

Sample size calculation

The required sample size was calculated using the formula

$N = \frac{Z_{\alpha}^{2}pq}{d^{2}}$

where Z_α_ represents the standard normal deviate corresponding to a 95% confidence interval (1.96), p represents the expected prevalence of diabetic autonomic neuropathy, q represents (100-p), and d represents the absolute precision. Based on a previous study conducted by Birajdar et al. [15], the prevalence of DAN was considered to be 58%. Using a confidence level of 95% and an absolute precision of 5%, the calculated sample size was 374. To ensure adequate recruitment and account for 5% non-response, the final target sample size was increased and rounded to 400 participants.

Sampling method

Wardhannapet taluk consists of 18 villages, of which three villages falling under the rural field practice area of Kakatiya Medical College - Wardhannapet, Bollikunta, and Panthini - were included in the study. The NCD register maintained at the primary health centres served as the sampling frame for the study. Each registered diabetic patient in the NCD clinic had a unique identification number. Using a random number table, diabetic patients were selected from the NCD register until the required sample size was achieved. The addresses of the selected individuals were obtained from the NCD clinic records and participants were visited in their respective households for data collection. If a selected participant was unavailable after two household visits, the next eligible participant from the same NCD register was approached to achieve the required sample size. A total of 409 patients with diabetes were approached, among whom 400 individuals provided informed consent and were included in the final study sample, while nine individuals declined to participate.

Study tool

Data were collected using a pre-tested semi-structured questionnaire. The questionnaire included information on socio-demographic characteristics, lifestyle factors such as smoking and alcohol consumption, dietary habits including fruit and vegetable intake, and the presence of co-morbid conditions. Anthropometric measurements and blood pressure were also recorded. Random blood glucose levels were measured for each participant using a digital glucometer during the field visit.

COMPASS-31 scale

Screening for DAN was carried out using the Composite Autonomic Symptom Score-31 (COMPASS-31) [14], a validated symptom-based questionnaire used for assessing autonomic dysfunction. The COMPASS-31 scale evaluates multiple domains of autonomic function including orthostatic intolerance, vasomotor, secretomotor, gastrointestinal, bladder, and pupillomotor functions. A cut-off score of ≥17 was used to identify participants with screen-positive autonomic dysfunction, based on the validation study by Greco et al. [16], which reported a sensitivity of 70% and specificity of 66.7% for the selected threshold. The COMPASS-31 score was used as a symptom-based screening measure and was not considered a definitive diagnostic test for DAN.

Data collection

Prior permission for the study was obtained from the Institutional Ethics Committee of Kakatiya Medical College. Data collection was carried out in the selected villages within the rural field practice area. Each participant was informed about the purpose of the study and written informed consent was obtained prior to participation. Participants were interviewed at their homes using the structured questionnaire. Questions were asked in the same sequence as listed in the questionnaire and sufficient time was given for participants to respond. Information obtained from participants was cross-checked with available medical records whenever possible. Confidentiality of participants and collected data was strictly maintained.

Data analysis

The collected data were coded and entered into Microsoft Excel 2013 (Microsoft, Redmond, WA) and analyzed using SPSS version 17.0 (SPSS Inc., Chicago, IL). Descriptive statistics such as frequencies and proportions were used to summarize the data. Variables showing a statistically significant association with DAN in the bivariate analysis (P<0.05) were entered into the multivariable binary logistic regression model. Clinically important variables considered potential confounders were also retained in the model irrespective of their statistical significance in the bivariate analysis.

## Results

A total of 400 participants were included in the study. Table 1 shows the socio-demographic characteristics of the study participants. The majority (176, 44.0%) were in the 45-59 years age group, and women constituted (220, 55.0%) of the study population. In this study, 172 (43.0%) participants were illiterate, and 192 (48.0%) were unemployed. Most participants (208, 52.0%) belonged to the lower socioeconomic class, while 192 (48.0%) were from the lower middle class.

**Table 1:** Socio‑demographic characteristics of the participants (N=400)

Variable	Category	Number (N)	Frequency (%)
Age group	30-44 years	124	31.0%
	45-59 years	176	44.0%
	≥60 years	100	25.0%
Gender	Male	180	45.0%
	Female	220	55.0%
Educational status	Illiterates	172	43.0%
	Less than primary	28	7.0%
	Primary school completed	52	13.0%
	Secondary school completed	88	22.0%
	High school completed	44	11.0%
	College	16	4.0%
Occupation	Unemployed	192	48.0%
	Unskilled	104	26.0%
	Semi‑skilled	68	17.0%
	Skilled	28	7.0%
	Semi‑professional	8	2.0%
Marital status	Married	396	99.0%
	Never married/Divorced/Widow	4	1.0%
Socio‑economic status	Lower middle class	192	48.0%
	Lower class	208	52.0%

Table 2 represents the clinical and behavioural characteristics of the participants. Regarding clinical characteristics, 156 (39.0%) participants had diabetes for less than five years, 140 (35.0%) had diabetes between five and 10 years and 104 (26.0%) had diabetes for more than 10 years. In terms of glycemic status, 248 (62.0%) participants had random blood sugar levels at ≥200 mg/dl, while 152 (38.0%) had levels <200 mg/dl. Hypertension was present in 248 (62.0%) participants, while 152 (38.0%) were normotensive. The vast majority (396, 99.0%) did not have leg ulcers. With respect to lifestyle factors, 64 (16.0%) participants were smokers, and 240 (60.0%) were alcoholics. Only 128 (32.0%) engaged in regular physical activity. Dietary assessment revealed that 200 (50.0%) consumed at least one serving of vegetables daily, whereas 376 (94.0%) did not consume at least one serving of fruits per day.

**Table 2:** Clinical and behavioural characteristics of the participants (N=400)

Variable	Category	Number (N)	Frequency (%)
Current smoker	Yes	64	16.0%
	No	336	84.0%
Current alcoholic	Yes	240	60.0%
	No	160	40.0%
Physical activity	Yes	128	32.0%
	No	272	68.0%
Daily vegetable intake	Yes	200	50.0%
	No	200	50.0%
Daily fruit intake	Yes	24	6.0%
	No	376	94.0%
Duration of diabetes	Less than 5 years	156	39.0%
	5-10 years	140	35.0%
	≥10 years	104	26.0%
Blood pressure	Hypertension	248	62.0%
	Normotensives	152	38.0%
Co-morbidities	Present	60	15.0%
	Absent	340	85.0%
Leg ulcers	Yes	4	1.0%
	No	396	99.0%
Random blood sugar levels	<200 mg/dl	152	38.0%
	≥200 mg/dl	248	62.0%

The overall prevalence of DAN among the study participants was 21.0% (84). Table 3 shows the association between socio-demographic variables and DAN. A higher proportion of DAN was observed among participants aged ≤44 years (52, 41.9%). With respect to gender, women had a higher prevalence of DAN (56, 25.4%). DAN was more common among illiterate participants (48, 27.9%) and the unemployed (52, 27.0%). Among smokers, 40 (62.5%) had DAN, compared to 44 (13.0%) among non-smokers. Similarly, 76 (31.6%) current alcohol consumers had DAN, whereas only eight (5.0%) non-alcoholics were affected. Dietary practices also influenced the prevalence of DAN. Participants who did not consume vegetables daily (52, 26.0%) and those not consuming fruits daily showed a higher prevalence (83, 22.0%). Physical inactivity was associated with a higher prevalence of DAN (66, 24.2%). With regard to clinical factors, participants with leg ulcers had a markedly higher prevalence of DAN (4, 50.0%). Additionally, those with a longer duration of diabetes (≥10 years) had a higher prevalence of DAN (28, 26.9%) compared to those with a duration of less than 10 years (56, 18.9%). Poor glycemic control was strongly associated with DAN, as 76 (30.6%) participants with random blood sugar levels ≥200 mg/dl had DAN, compared to only eight (5.2%) among those with levels <200 mg/dl.

**Table 3:** Association between socio-demographic factors and diabetic autonomic neuropathy (DAN) (N=400) *P<0.05, statistically significant.

Variable	Category	DAN Yes n (%)	DAN No n (%)	P value
Age group	30-44	52 (41.9%)	72 (58.1%)	<0.0001*
	45-59	20 (11.3%)	156 (88.7%)	
	≥60	12 (12.0%)	88 (88.0%)	
Gender	Male	28 (15.5%)	152 (84.4%)	0.015*
	Female	56 (25.4%)	164 (74.5%)	
Educational status	Illiterates	48 (27.9%)	124 (72.0%)	0.013*
	Less than primary school	9 (32.1%)	19 (67.8%)	
	Primary school completed	8 (15.3%)	44 (84.6%)	
	Secondary school completed	12 (13.6%)	76 (86.3%)	
	High school completed	6 (13.6%)	38 (86.3%)	
	College	1 (6.2%)	15 (93.7%)	
Occupation	Unemployed	52 (27.0%)	140 (72.9%)	0.0009*
	Unskilled	20 (19.2%)	84 (80.7%)	
	Semi-skilled	4 (5.8%)	64 (94.1%)	
	Skilled	4 (14.2%)	24 (85.7%)	
	Semi-professional	4 (50.0%)	4 (50.0%)	
Marital status	Married	83 (20.9%)	313 (79.0%)	0.843
	Never married/divorced/widow	1 (25.0%)	3 (75.0%)	
Socio-economic status	Lower middle class	39 (20.3%)	153 (79.6%)	0.745
	Lower class	45 (21.6%)	163 (78.3%)	

Table 4 shows the binary logistic regression analysis of factors associated with DAN. Binary logistic regression showed that factors such as age ≤44 years (COR: 4.81 (95% confidence interval (CI): 1.99-6.65), p<0.001), women (COR: 1.85 (95%CI: 1.13-3.04), p=0.015), illiteracy (COR: 2.06(95%CI: 1.28-3.32), p=0.003) and unemployment (COR: 2.04 (95%CI: 1.26-3.32), p=0.004) led to significantly higher odds of DAN. Specifically, current smokers (COR: 11.06 (95%CI: 2.86-12.80), p<0.001), alcohol consumption (COR: 8.81 (95%CI: 2.59-10.00 ), p<0.001), participants not consuming vegetables (COR: 1.85(95%CI: 1.14-2.99), p=0.012 and physical inactivity (COR: 1.96(95%CI: 1.12-3.42), p=0.018) also had significantly higher odds of DAN compared to their counterparts. Among clinical variables, hypertension (COR: 0.99(95%CI: 0.59-1.67), p=0.97) and duration of diabetes ≥10 years (COR: 1.58(95%CI: 0.96-2.59), p=0.07) led to higher odds of DAN; however, the association was not statistically significant. Participants with leg ulcers had higher odds of DAN (COR: 3.90 (95%CI: 1.00-5.21)), with borderline statistical significance (p=0.05). Poor glycemic control demonstrated a strong and statistically significant association with DAN. Participants with random blood sugar levels ≥200 mg/dl had significantly higher odds of DAN compared to those with levels <200 mg/dl (COR: 7.97(95%CI: 2.48-10.65), p<0.001).

**Table 4:** Univariate binary logistic regression analysis of factors associated with diabetic autonomic neuropathy (DAN) among study participants *P<0.05, statistically significant. DAN: Diabetic autonomic neuropathy; DM: diabetes mellitus; RBS: random blood sugar; COR: crude odds ratio.

Variable	Category	DAN +	DAN -	COR (95%CI)	P value
Age	≤44 years	52(41.9%)	72(58.0%)	4.81 (1.99-6.65)	<0.001*
	≥45 years	32(11.5%)	244(88.4%)		
Gender	Female	56 (25.4%)	164 (74.5%)	1.85 (1.13-3.04)	0.015*
	Male	28 (15.5%)	152 (84.4%)		
Educational status	Illiterate	48(27.9%)	124(72.0%)	2.06 (1.28-3.32)	0.003*
	Literate	36(15.7%)	192(84.2%)		
Occupation	Unemployed	52(27.0%)	140(72.9%)	2.04 (1.26-3.32)	0.004*
	Employed	32(15.3%)	176(84.6%)		
Current smokers	Yes	40 (62.5%)	24 (37.5%)	11.06 (2.86-12.80)	<0.001*
	No	44(13.0%)	292 (86.9%)		
Current alcoholics	Yes	76 (31.6%)	164 (68.3%)	8.81 (2.59-10.00 )	<0.001*
	No	8 (5.0%)	152 (95.0%)		
Daily veg intake	Yes	32 (16.0%)	168 (84.0%)	1.85 (1.14-2.99)	0.012*
	No	52 (26.0%)	148 (74.0%)		
Daily fruit intake	Yes	1 (4.1%)	23 (95.9%)	6.52 (0.85-10.91)	0.071
	No	83 (22.0%)	293 (78.0%)		
Physical activity	Yes	18(14.0%)	110 (85.9%)	1.96 (1.12-3.42)	0.018*
	No	66 (24.2%)	206 (75.7%)		
Hypertension	Hypertensives	52 (20.9%)	196 (79.0%)	0.99 (0.59-1.67)	0.97
	Normotensives	32(21.0%)	120 (78.9%)		
Duration of DM	<10 yrs	56(18.9%)	240(81.0%)	1.58 (0.96-2.59)	0.07
	≥10 yrs	28(26.9%)	76(73.0%)		
Leg ulcer	Yes	4 (50.0%)	4 (50.0%)	3.90 (1.00-5.21)	0.05
	No	80 (20.4%)	312 (79.5%)		
RBS	<200 mg/dl	8 (5.2%)	144 (94.7%)	7.97 (2.48-10.65)	<0.001*
	≥200 mg/dl	76 (30.6%)	172 (69.3%)		

Table 5 indicates the results of multivariate logistic regression analysis performed to identify independent factors associated with DAN. Age remained a statistically significant independent predictor, with participants aged ≤44 years demonstrating higher odds of DAN compared to those aged ≥45 years (adjusted odds ratio (AOR): 3.10 (95%CI: 1.58-6.08); p=0.001), women (AOR: 1.72 (95%CI: 1.01-2.92); p=0.045), illiteracy (AOR: 1.85 (95% CI: 1.08-3.16); p=0.024), unemployment (AOR: 1.67 (95%CI: 1.01-2.76); p=0.046), smoking (AOR: 7.95 (95%CI: 2.31-10.33); p=0.001), alcohol consumption (AOR: 5.21 (95%CI: 1.95-8.93); p=0.001), not consuming vegetables daily (AOR: 1.64 (95%CI: 1.01-2.66); p=0.045), physical inactivity (AOR: 1.72 (95%CI: 1.00-2.95); p=0.049) and poor glycemic control (AOR: 6.03 (95%CI: 2.07-7.58); p=0.001) were identified as significant independent factors associated with DAN.

**Table 5:** Multivariable binary logistic regression analysis of factors associated with DAN among study participants *P<0.05, statistically significant. DAN: Diabetic autonomic neuropathy; AOR: adjusted odds ratio.

Variable	Category	DAN +	DAN -	AOR (95% CI)	P value
Age	≤44 years	52(41.9%)	72(58.0%)	3.10 (1.58-6.08)	0.001*
	≥45 years	32(11.5%)	244(88.4%)		
Gender	Female	56 (25.4%)	164 (74.5%)	1.72 (1.01-2.92)	0.045*
	Male	28 (15.5%)	152 (84.4%)		
Educational status	Illiterate	48(27.9%)	124(72.0%)	1.85 (1.08-3.16)	0.024*
	Literate	36(15.7%)	192(84.2%)		
Occupation	Unemployed	52(27.0%)	140(72.9%)	1.67 (1.01-2.76)	0.046*
	Employed	32(15.3%)	176(84.6%)		
Current smokers	Yes	40 (62.5%)	24 (37.5%)	7.95 (2.31-10.33)	0.001*
	No	44(13.0%)	292 (86.9%)		
Daily veg intake	Yes	32 (16.0%)	168 (84.0%)	1.64 (1.01-2.66)	0.045*
	No	52 (26.0%)	148 (74.0%)		
Physical activity	Yes	18(14.0%)	110 (85.9%)	1.72 (1.00-2.95)	0.049*
	No	66 (24.2%)	206 (75.7%)		
RBS	<200 mg/dl	8 (5.2%)	144 (94.7%)	6.03 (2.07-7.58)	0.001*
	≥200 mg/dl	76 (30.6%)	172 (69.3%)		
Current alcoholics	Yes	76 (31.6%)	164 (68.3%)	5.21 (1.95-8.93)	0.001*
	No	8 (5.0%)	152 (95.0%)		

## Discussion

This cross-sectional study estimated the prevalence and factors associated with DAN among patients with diabetes in rural Telangana. The prevalence of DAN among 400 individuals with diabetes mellitus was found to be 21.0% (84). This finding is comparable to the study from Riyadh by AlOlaiwi et al. [17]. However, the prevalence observed in the current study is considerably lower than hospital-based studies reported from Maharashtra and Karnataka by Birajdar et al. and Subraya and Mahesh [15,18]. The relatively lower prevalence in the present study may be attributed to differences in study settings. Being community-based, the present study likely included individuals across a broader spectrum of disease severity, whereas hospital-based studies tend to include patients with more advanced disease and complications, thereby overestimating prevalence. Additionally, variations in diagnostic criteria and assessment methods used for identifying DAN could have contributed to the observed differences. Furthermore, a study conducted in Puducherry by Thekkur et al. [19] among 293 diabetic patients reported a lower prevalence of 10.6% (25 participants). The discrepancy between these findings and the present study may be explained by geographical, cultural, and lifestyle differences, as well as variations in the study population.

In the present study, DAN showed a significant association with several sociodemographic, lifestyle, and clinical factors. Younger age (≤44 years), women, illiteracy, unemployment, alcohol consumption, smoking, physical inactivity, presence of leg ulcers, and poor glycemic control were found to be significantly associated with the occurrence of DAN.

In the present study, younger age demonstrated significantly higher odds of DAN. This finding contrasts with studies in which increasing age was reported as an important factor associated with autonomic dysfunction. This difference observed in the present study may be due to the epidemiological transition in India, leading to early onset of type 2 diabetes mellitus and early development of complications. In the current study, female gender was significantly associated with DAN. A meta-analysis [20] reported female gender to be an independent risk factor for diabetic neuropathies. This association may reflect differences in healthcare-seeking behaviour and other unmeasured factors. However, these findings were not consistent with the study by Agarwal et al. [21]. 

In the present study, illiteracy and unemployment were associated with significantly higher odds of DAN. These findings were consistent with the study by Thekkur et al. [19]. Illiteracy and unemployment, being important social determinants of health, may impair disease awareness and treatment adherence, leading to poor glycemic control and a higher risk of complications [22]. The study revealed that lifestyle factors such as smoking, alcohol consumption, and physical inactivity were significantly associated with increased odds of DAN. These emerged as the strongest independent factors associated with DAN. The findings were consistent with previous literature. Lifestyle factors are key modifiable determinants linked to insulin resistance and diabetes-related complications [23]. Among the clinical variables, poor glycemic control was associated with significantly increased odds of DAN, which is consistent with the Chennai Urban Rural Epidemiology Study (CURES) study [24] published in 2023. In the present study, hypertension and longer duration of diabetes demonstrated higher odds of DAN; however, the association was not statistically significant. In contrast, Agarwal et al. [21] reported a significant association between hypertension, longer duration of diabetes, and DAN.

Our study adds to the existing literature by specifically focusing on a rural population in Telangana, an underrepresented group in research on DAN. The community-based design and inclusion of a relatively large sample of participants enhance the representativeness and reliability of the findings. The use of a validated non-invasive questionnaire for the assessment of autonomic neuropathy ensures uniformity and feasibility in field settings. Furthermore, by analyzing a comprehensive range of sociodemographic, lifestyle, and clinical factors and adjusting for potential confounders through multivariate analysis, the study provides a more accurate identification of independent factors associated with DAN.

The cross-sectional design limits the ability to establish a causal relationship between the identified risk factors and DAN. The study was conducted in a rural setting in Telangana, which may limit the generalizability of the findings to urban populations or other regions. The assessment of lifestyle factors such as smoking, alcohol consumption, and physical activity was based on self-reported data and is therefore subject to recall and social desirability bias. Important factors such as medication adherence and psychological factors were not included in the study, which might have provided additional insights into the risk factors for DAN. Additionally, COMPASS-31 is a symptom-based screening instrument and does not constitute definitive autonomic function testing or a clinical diagnosis of diabetic autonomic neuropathy. Therefore, the prevalence observed in this study should be interpreted as screen-positive autonomic dysfunction based on the selected COMPASS-31 threshold. When the selected participants were not available even after two visits, the next eligible participant from the sampling frame was selected. This might have caused selection bias.

## Conclusions

In the present study, approximately one in five individuals with type 2 diabetes mellitus screened positive for autonomic dysfunction based on the COMPASS-31 threshold. Younger age, female sex, lower educational status, unemployment, current smoking, inadequate vegetable intake, physical inactivity, elevated random blood glucose, and current alcohol use were independently associated with DAN. Given the cross-sectional nature of the study, these associations should not be interpreted as causal. Further longitudinal studies are warranted to establish temporal relationships between these factors and DAN. Community-based screening and appropriate clinical assessment may help facilitate early identification of autonomic symptoms among individuals with type 2 diabetes in rural settings.
